# Sources of teacher efficacy in teaching reading: success, sharing, and support

**DOI:** 10.1007/s44020-022-00016-0

**Published:** 2022-12-12

**Authors:** Mary Ryan, Graham D. Hendry

**Affiliations:** 1 Catholic Schools New South Wales, Sydney, Australia; 2 Centre for Educational Measurement and Assessment, Sydney School of Education and Social Work, Faculty of Arts and Social Sciences, University of Sydney, Room 328, Education Building A35, Sydney, NSW 2006 Australia

**Keywords:** Teacher efficacy, Self-efficacy, Collective efficacy, Teaching, Reading

## Abstract

Teacher self- and collective efficacy are important motivational factors in determining teachers’ effort in their professional practice. Teachers’ higher levels of self- and collective efficacy are positively associated with student achievement in specific domains, and teachers’ enhanced wellbeing and commitment to their profession. This qualitative study explores primary teachers’ own views about naturalistic and characteristic experiences and influences that have strengthened and weakened their efficacy in teaching reading. Five themes of naturalistic sources of enhancement in self- and collective efficacy are identified that are consistent with predictions of Bandura’s social cognitive theory and studies involving interventions in schools by expert trainers. The themes are combined into a model of growth in teachers’ efficacy and can potentially inform decision-making in schools in supporting and enhancing teachers’ self- and collective efficacy when teaching reading.

## Introduction

The effort that teachers make in their professional practice is determined by many motivational factors. The most fundamental of these factors is teachers’ moral obligation, or duty, to care for students and help them to learn. Other important motivational factors include teachers’ confidence in their professional capabilities, or what is called their self-efficacy in various areas of practice (e.g. teaching reading) (Raymond-West & Snodgrass Rangel, 2020; Tschannen-Moran & Johnson, 2011). The term “self-efficacy” was coined by Albert Bandura in his social cognitive theory of human agency to refer to a person’s belief in their capability, or ability to successfully perform and be effective, in a certain situation (Bandura, 1997, 2006). According to Bandura’s theory (1989; 1997), teachers’ strength or level of self-efficacy—in a particular teaching situation—contributes to determining the level of effort that they make, how long they persist with that effort, and how resilient they are in the face of difficulties in that situation (Morris et al., 2017; Tschannen-Moran & Johnson, 2011).

Bandura (1997) also proposes that when a person is in a group or organisation, their personal self-efficacy is influenced by the strength of beliefs that they have in the capability of people around them. While teachers may often practice alone in their classroom, they are also members of teaching teams and a school organisation. Teachers’ strength of beliefs about their colleagues’ and/or whole school to be successful and effective en masse is called *collective efficacy* (Goddard et al., 2000); it is “the perceptions of teachers in a school that the efforts of the faculty as a whole will have a positive effect on students” (p. 480).

Research has examined teacher efficacy both *overall* and in specific teaching situations or areas of practice, also called *domains*, and has shown a clear positive relationship between teachers’ level of self-efficacy and the quality of students’ learning. Students taught by teachers with higher self-efficacy (in particular areas of practice) have higher levels of achievement, motivation, positive attitudes, and self-efficacy (Skaalvik & Skaalvik, 2007). Teachers with higher levels of self-efficacy overall also have enhanced well-being and are less likely to leave the profession or “burn out” (feel emotionally and physically exhausted because of stress) (Morris et al., 2017; Skaalvik & Skaalvik, 2007; Zee & Koomen, 2016).

Teachers’ collective efficacy is also positively related to students’ achievement (Tschannen-Moran & Barr, 2004). Higher collective efficacy is associated with teachers’ increased leadership, more comprehensive implementation of school-wide strategies, and more positive attitudes to professional learning (Donohoo, 2018). Skaalvik and Skaalvik (2007) also found that teachers’ self- and collective efficacy are positively related.

Given the positive relationship between teacher self- and collective efficacy and students’ achievement, and teachers’ well-being, it is vital in education to understand the sources of enhancement in teachers’ efficacy. Fundamentally, a teacher’s strengthened belief in their capability sustains their moral obligation, while a teacher’s weakened efficacy could lead them to experience psychological and *moral* distress (Afsar et al., 2019; Morley et al., 2019).

## Influences on teacher efficacy

Bandura’s social cognitive theory predicts that self-efficacy is strengthened the most when people experience *enactive mastery*, or success in their genuine attempts to do things, and they have what are called *mastery experiences* (Bandura, 1989). Self-efficacy is also strengthened, although not as fully, through vicarious experience when a person observes someone else engaged in successful performance (this is called observational learning or *modelling*). Being verbally persuaded that they can master something also strengthens a person’s self-efficacy, although this is presumed to be less effective than modelling; and finally, from positively interpreting their state of arousal in challenging situations, people can also enhance their self-efficacy (Bandura, 1989).

Several authors have proposed that teachers’ collective efficacy is enhanced in similar ways to their personal self-efficacy. For example, collective efficacy may be strengthened when teachers in a school experience genuine success in their collaborative endeavours (enactive mastery); hear about or observe firsthand successful achievements by teaching teams in other schools (modelling); and/or are verbally persuaded together in social situations, e.g. during talks by school leaders and/or facilitators in professional learning programmes (Goddard et al., 2000; Tschannen-Moran & Barr, 2004).

However, despite considerable research since the 1970s on the development of teacher self-efficacy measures and examination of relationships between scores on these measures and other variables, we still know relatively little about naturalistic and characteristic sources of enhancement in teacher efficacy (Klassen et al., 2011). In the only qualitative study that has investigated teachers’ views of naturalistic sources of *overall* self-efficacy for teaching, Wang et al. (2017) found that Singaporean secondary teachers thought their self-efficacy was strengthened by mastery experiences in helping students to “improve their academic performance and … produce good exam results” (p. 143). Teachers also thought their self-efficacy was strengthened by observing colleagues teach successfully, and being “verbally persuaded” by their school leaders, colleagues and students. Verbal persuasion included being “appreciated and validated” by leaders, “praised” by colleagues, and “valued and appreciated” by students. These findings confirm predictions of Bandura’s social cognitive theory about sources of enhancement in teachers’ self-efficacy. Wang et al. (2017) also found that some teachers thought that their self-efficacy was strengthened by having in-depth knowledge of their students’ backgrounds, rapport with their students, and/or work experience outside of teaching.

With respect to specific *domains* of teacher self-efficacy, in a qualitative study of naturalistic sources of enhancement in self-efficacy in teaching using mobile devices (mini-iPads), Tilton and Hartnett (2016) found that teachers’ self-efficacy was strengthened over 1 year through their experiences of success (enactive mastery) in using mini-iPads in their classrooms, and seeing successful use of the devices modelled by colleagues. In a recent study of teacher self-efficacy in another domain—teaching reading (Clark, 2020)—a positive correlation was found for the first time between self-efficacy and a teacher having a teacher mentor. Presumably, for the mentored teachers (who had only been teaching for 1 year), their mentor modelled good practice and/or persuaded them that they could be successful. As the author notes, the statistical “analysis only provides initial answers and more research is needed” (p. 139). In another study of self-efficacy in teaching reading with early career teachers’ (teachers with less than 2-years’ experience), Raymond-West and Snodgrass Rangel (2020) found a positive correlation between teachers’ self-efficacy and their level of “exposure” to literacy instruction in their pre-service “field experiences”. However, it is unclear from this study whether teachers’ pre-service field experiences included enactive mastery, modelling or verbal persuasion, or all three in a range of combinations.

More research is also needed on naturalistic sources of teacher collective efficacy (Abedini et al., 2018). In the only study on teachers’ *own views*, Loughland and Ryan (2022) found that aspiring school leaders thought sources of collective efficacy included professional learning programmes that led to teachers being successful in their practices (enactive mastery); leaders’ own experience of observing successful leadership in other schools (modelling); effective communication and collaboration between teachers; and leaders and teachers showing respect for and trust in each other. These findings are reinforced somewhat by a study of the effects of a professional learning programme on primary teachers’ literacy instruction and assessment (Ciampa & Gallagher, 2016). This “job-embedded, school-based” programme involved teachers in collaboratively co-planning instruction and participating in classroom observations over several months. Ciampa and Gallagher found that teachers’ overall efficacy was enhanced by collaboration that resulted in successful outcomes (enactive mastery); observation of each other’s teaching (modelling); and “coaching” by colleagues and external “literacy coaches”. Coaching included giving advice and feedback. In another study of the effects of a school-based (year-long) professional learning programme on teacher’s efficacy in literacy instruction, Chambers Cantrell and Hughes (2008) found that the programme enhanced teachers’ self-efficacy primarily through ongoing support provided by external “coaches” who had expertise in literacy instruction. Coaches supported teachers “by modelling strategies, providing resources according to perceived need and upon the request of teachers, observing lessons, providing feedback, and assisting lesson planning” (p. 115). Chambers Cantrell and Hughes also suggest that the collaborative nature of the professional learning programme enhanced teachers’ *collective efficacy* by strengthening “teachers’ sense that their faculties could influence students’ literacy achievement” (p. 119).

In sum, studies of school-based professional learning programmes in literacy instruction, and the few studies on naturalistic and characteristic sources of enhancement in teacher efficacy appear to confirm the predictions of Bandura’s theory, but more research, particularly in important areas of practice, is needed. As Klassen et al. (2011) argue, in the past, “insufficient attention has been paid to the sources of teachers’ self- and collective efficacy” (p. 31), while Morris et al. (2017), in their critical review, conclude that future research on the “precursors” of teacher self-efficacy needs to concentrate on exploratory studies.

## Context of our study

Correspondingly, our qualitative study explores how teachers think that they have developed their self- and collective efficacy in the important domain of *teaching reading*. The ability to read enables people to engage in education, acquire knowledge, and participate fully in society, and effective literacy instruction in primary (Kindergarten to Year 6) schools is crucial for children to learn to read (Castles et al., 2018; National Inquiry into the Teaching of Literacy (Australia), 2005). One of us is a manager in the governing body Catholic Schools New South Wales, which contributes to teacher professional learning projects in schools in New South Wales (NSW) regional and rural areas. Our association with Catholic Schools New South Wales gave us an opportunity to recruit teachers from primary schools in regional and rural areas for our study.

Bandura’s theory also frames our inquiry, and while we seek to test predictions of the theory, we are open to exploring teachers’ own perspectives. For example, in a qualitative study of the influence of school principals’ actions on *secondary teachers’ emotions*, Lambersky (2016) found incidentally that teachers perceived their self-efficacy was enhanced when their principal showed them respect for their “professional” capabilities (e.g. in preparing lessons).

Our research questions are as follows:

1. What experiences or influences do primary teachers think have led to them developing their self-efficacy in teaching reading?
2. What experiences or influences do primary teachers think have led to them developing their collective efficacy in teaching reading?

## Method

This study was approved by the Human Research Ethics Committee of the University of Sydney (protocol number 2021/213). Participants gave informed consent. The research method adopted for this study was semi-structured individual interviews. The semi-structured interview is a flexible method, allowing an interviewer to ask not only standard questions to ensure consistency, but also probing questions to clarify the meanings that interviewees give to their experiences (Barriball & While, 1994; Lambersky, 2016). Within the literature on qualitative methods, at least eight interviewees are considered a satisfactory sample size for a robust study (Baker & Edwards, 2014; McCracken, 1988). Our interview sample comprised 12 volunteer teachers, and included a minimum of two teachers from each of five (out of a total of 19) primary schools in the Wilcannia-Forbes diocese in western (regional and rural) NSW, Australia. Teachers varied in their teaching experience, which we defined as the number of years they had been teaching.

Interviews were conducted face-to-face by the first author at teachers’ schools in a private room at the school. All NSW government COVID-19 (coronavirus) restrictions at the time for regional and rural NSW (e.g. maintaining 1.5-m physical distancing) were strictly complied with. Standard interview questions included the following: “Can you please tell me about a time when you have been successful in teaching reading?”, “How did this experience influence your confidence (if at all) in your ability to teach reading?”, “What other experiences or influences do you think have made you feel confident in your ability to teach reading?”, and “What influences do you think schools as a whole have had on your confidence or belief in your ability to teach reading?”. We also asked teachers about experiences or influences (if any) that they thought had made them feel *less* confident in their ability to teach reading.

The average duration of interviews was 45 min. Each interview was audio recorded with the participant’s consent and recordings were professionally transcribed. We used the qualitative technique of thematic analysis (Braun & Clarke, 2006; Miles & Huberman, 1994) to analyse each transcript, with the aim of identifying key experiences and themes in teachers’ views in relation to each of our two research questions.

In the initial phase of the analysis, we independently read the transcripts to gain familiarity with and become immersed in the data. In the second phase, initial codes were generated by “coding interesting features of the data in a systematic fashion across the entire data set, collating data relevant to each code” (Braun & Clarke, 2006, p. 87). In the third phase, potential and emerging themes were identified. For example, in relation to our first research question, we generated codes such as, “Seeing children’s success” and “Children being successful” from our data. Examples from our data included “I see the children being successful”; “I see the child achieving”; “I could see that the kids (were reading)”; “Seeing I guess their success”; and “I could see that kids were getting so much out of it”. From these codes emerged one of our main themes (see below). In the fourth phase, the main themes and sub-themes were defined and reviewed, and we discussed and confirmed whether they were representative of the codes and the entire data set. In the final phase, the essence of each theme was clarified, and each theme was named.

## Results

The number of years teachers in our sample had been teaching ranged from less than 3 to more than 20 years. For the purpose of maintaining confidentiality, when we include quotes of teachers’ words in the following sections, we call teaching experience that is less than 5 years, “early career”, and more than 5 years, “experienced”. Overall, early career teachers had taught across less primary years or grades (Kindergarten, Year 1, etc.) than experienced teachers.

Five themes emerged in relation to each of our two research questions. Each theme is described below under each question. The five themes are also listed against each research question in Table 1.

**Table 1:** Research questions and associated themes

Research question	Themes			
*1. What experiences or influences do primary teachers think have led to them developing their self-efficacy in teaching reading?*	Observing children’s success	Sharing practice: Knowledge and modelling	Leadership support	Professional learning: New knowledge and skill
*2. What experiences or influences do primary teachers think have led to them developing their collective efficacy in teaching reading?*	Whole-school culture			

### What experiences or influences do primary teachers think have led to them developing their self-efficacy in teaching reading?

#### Observing children’s success

Without exception, teachers thought that observing children’s success in reading as a result of their help or teaching, either on a specific occasion or over longer periods, was an important influence that enhanced their confidence in their ability to teach reading. This was particularly the case when teachers helped children who were struggling to read to be successful; some teachers described such experiences as “powerful”.

Teachers used a variety of systematic strategies and reading programmes to help children to learn to read. Observing children’s success affirmed for teachers that the strategies they were using were working:

If I’m seeing [children’s] success, then I feel like I’m successful … It spurs you on to keep going because … you’re seeing progress through there and you’re thinking yes, what I’m doing is working here. (Teacher One, experienced)

The moment I started introducing these decodable texts, I felt a moment of success. Because I could see that the kids were – they weren’t looking at the pictures to read the text … They were sounding out their letters and they were reading the words by learning those sounds … I felt so much more confident going into my morning block and teaching my guided reading. (Teacher Two, early career)

In teachers’ experience, children’s success took a variety of forms; typically, teachers could see children were successful when they could read or were able to meet certain benchmarks or measurement standards (e.g. could clearly pronounce certain sounds). Teachers could also see children were successful when they were engaged or engrossed, and expressed happiness, enjoyment, or “joy” in reading; wanted to read more; or were “proud” of their reading achievements.

For one teacher, observing children’s enjoyment in and increased motivation for reading was more important for enhancing their self-efficacy in teaching reading than seeing the results of standardised measures of achievement (e.g., data from a national test of literacy).

It gave me the confidence because I could see … that kids were getting so much out of [my strategy] and that was more than … the data, more than the measurable side of things. (Teacher Nine, experienced)

Observing children’s success in reading also motivated teachers to persist in their endeavours to help children, particularly those who were struggling to learn to read.

#### Sharing practice: knowledge and modelling

Teachers thought that colleagues sharing their practice in a supportive way was an important influence that enhanced their confidence in their ability to teach reading. The main purpose of sharing practice was to help a colleague to improve their skills and overcome challenges, particularly in their early years of teaching. Supportive sharing of practice took two interrelated forms.

First, it involved colleagues in generously sharing their knowledge about and suggesting strategies for teaching reading, and/or sharing resources (e.g. a book) about such strategies:

The teacher I team taught with in my first official year of teaching was amazing and so supportive … that teacher was happy to share ideas and resources and gave me a lot of information in my first year out. (Teacher Six, experienced)

Second, sharing practice also involved colleagues, who were successful in their teaching, modelling, or demonstrating strategies in real time in the classroom for their peers:

She modelled with me for a week. So, we sat at my guided reading table, and she took my groups for a week while I watched. Then the following week, she sat next to me, and I did it. Then she was like, “You don’t need me anymore”. (Teacher Two, early career)

In one case, a colleague was also videoed modelling strategies and peers watched the video later.

#### Leadership support

Teachers also thought that, in particular, support provided by their school leaders (who included principals and assistant principals) enhanced their confidence in their ability to teach reading. Similar to sharing practice, leadership support took two interrelated forms.

First, it involved principals or assistant principals having time for and being open to listening to teachers’ requests for help to overcome challenges they were experiencing, then suggesting specific reading programmes or systematic strategies. In small schools, teaching principals would often also model these strategies for their teachers.

[Name] has been a huge support as a principal … having never taught kinder before, either, it was yeah, very daunting [for me]. But [my principal] would come in and model what [they] used to do when [they were] in kinder … I was always happy for [them] [to do that]. (Teacher Four, early career)

Second, leadership support also involved principals in interpersonal interactions showing trust in their staff and belief that they could be successful in teaching reading:

That’s important for me, really important for me … having [a] principal who just sort of said straight out, “I believe that you can do this”, really helped. (Teacher Five, experienced)

#### Professional learning: new knowledge and skill

Some teachers were also of the view that professional learning experiences (e.g., promoted by their principal) that led them to develop new knowledge and skill for teaching reading also enhanced their confidence in their ability to teach reading. Teachers valued learning about new reading programmes or systematic strategies that enabled them to be successful:

So having the experience of [two reading programmes] … I’ve learnt a lot more about [teaching reading] … through those programmes, I’ve learnt more about the systematic approach to things and so the experience of using them and having them [be] successful has made me say, “Well, yes, I will persevere with this”. (Teacher Seven, experienced)

In some cases, as with teachers sharing their practice for their colleagues in supportive ways, professional learning facilitators who visited a school also modelled or demonstrated strategies in the classroom for participants. For some teachers, learning about a new reading programme also enhanced their confidence because it affirmed their current thinking and practices.

#### Influences that made teachers feel less confident in their ability to teach reading

Teachers identified a variety of individualised influences that made them feel less confident in teaching reading, which in general related conversely to positive influences on teachers’ self-efficacy. Experiences that made some teachers feel less confident or made them doubt their capability included not being able to help a child (particularly a child with high needs) make progress in their reading. Some teachers also experienced less confidence because of a requirement and/or expectation (e.g. from a principal) to frequently collect test data about students’ achievements, but then the data does not show growth in students’ skill, and yet the teacher sees that children are being successful in their own unique ways.

I think sometimes [there are] too many expectations so far as tracking and assessment and data … the little child in the class picking up the book or looking at the words or knowing five letters … is the achievement [but] … data can get in the way and it can make you feel like … maybe [you’re] not making an impact. (Teacher One, experienced)

Other experiences that made some teachers feel less confident in teaching reading included being peer reviewed in their teaching by a person that they had no rapport with. Some teachers also thought that being spoken to in a disrespectful way by their principal (e.g. about students’ test results) weakened their confidence. For one teacher, this experience had a profoundly negative effect on their self-efficacy and willingness to try new strategies.

Some teachers were also of the view that when specific reading programmes or systematic strategies for teaching reading were introduced to them (e.g. by a principal) without explanation about why they should use them, and/or without teachers being able to easily relate such programmes or strategies to their current practices, then this experience also weakened their confidence in their ability to teach reading.

### What experiences or influences do primary teachers think have led to them developing their collective efficacy in teaching reading?

#### Whole-school culture

For teachers at some schools, a whole-school culture of a collaborative approach to teaching reading in their school, in which all their colleagues taught in the same, accepted and successful ways (e.g. by using the same reading programme), was an important influence that enhanced their confidence in their ability to teach reading:

That just gave me such confidence, working with that [programme] and having my colleagues do the same thing. We all put our hands up and said we’ll do it. (Teacher Nine, experienced)

This whole-school culture evolved from teachers engaging in professional learning—promoted by their leadership team—and attempting to implement new teaching strategies. The culture also evolved from leaders communicating their encouragement to, and showing trust in, their teachers’ to make judgements and decisions, so that teachers felt “safe” in attempting new strategies:

[Our principal] make[s] it feel like a safe place where – and if you make a mistake that’s okay [our principal] has given us lots of opportunity … to try different things in our classrooms. To have that freedom to say, we’re going to give this a go. (Teacher Eight, early career)

In one school, where the teaching principal also modelled strategies, this feeling of safety extended to all teachers collaborating and feeling comfortable to share their practice by modelling strategies for their colleagues.

#### Discussion

This qualitative study explored 12 primary teachers’ views about experiences or influences that they thought had led to them developing their self- and collective efficacy in teaching reading. This study also explored primary teachers’ views about influences that made them feel less confident in teaching reading. Our qualitative results showed that the most naturalistic and characteristic source of enhancement in teachers’ self-efficacy (in teaching reading) was observing the success that children had in reading. This source of enhancement was powerful when teachers helped children who were experiencing greater difficulty learning to read. This finding confirms the prediction of Bandura’s social cognitive theory that self-efficacy (in a specific domain) is strengthened when people have enactive mastery experiences (experience success in their genuine attempts to do things). This finding also reinforces Tschannen-Moran and Johnson’s (2011) claim that teachers’ self-efficacy is enhanced by “teaching accomplishments with students” (p. 752). Our results are consistent with those of a quasi-experimental study in which teachers’ self-efficacy in teaching reading was enhanced following a specific mastery experience that involved teachers using a hand-signing strategy to teach sounds of the alphabet. In implementing the strategy, teachers participated with an external “trainer” in almost 6 h of professional learning that included the trainer modelling practice in teachers’ classrooms and giving individualised verbal persuasion (Tschannen-Moran & McMaster, 2009).

While teachers in our study did not specifically mention being “persuaded” as a source of enhancement in their self-efficacy in teaching reading, our results show that school leaders’ support, which includes, e.g. principals giving teachers encouragement and showing belief in their capabilities, is a naturalistic source of enhancement. This source could be interpreted as simple “verbal persuasion”, but we suggest that it is more usefully interpreted as *mentoring* characterised by *autonomy support*, which is defined as teachers’ interpersonal, nurturing behaviour (Katz & Shahar, 2015; Reeve, 2009) that includes adopting students’ perspectives, explaining the rationale for tasks, and showing “patience to allow time for self-paced learning” (Reeve, 2009, p. 106). In the same way, we suggest that supportive leaders referred to by teachers in our study were likely to adopt the perspective of their staff, explain the rationale for a new teaching strategy or reading programme, and show patience with their teachers’ attempts at implementation. As one teacher who felt supported commented, “[My principal] doesn’t say, ‘I want you to implement it tomorrow’. [They] give you time to try the new thing” (Teacher Five, experienced). It could be that it was this form of mentoring that Clark (2020) found was positively correlated with reading teachers’ self-efficacy.

Consistent with previous research (Outlaw & Grifenhagen, 2021; Tschannen-Moran & McMaster, 2009), our results show that professional learning for developing new knowledge and skill in implementing a new systematic strategy or programme for teaching reading is a source of enhancement in teachers’ self-efficacy. Our results also show that teachers’ sharing of practice that includes colleagues openly demonstrating their successful teaching actions for peers is a naturalistic source of enhancement in teachers’ self-efficacy. This source confirms the prediction of Bandura’s theory that self-efficacy is strengthened through vicarious experience when a person observes others performing successfully. As one teacher commented about their experience of observing a colleague teaching reading well, “if [they] can do that, I can do it too” (Teacher One, experienced). The importance of modelling is reinforced by research in which pre-service teachers’ self-efficacy in literacy instruction was “positively influenced” by experienced teachers modelling practice (Tschannen-Moran & Johnson, 2011), and a study in which early-career teachers in schools in predominately rural areas strengthened their self-efficacy when they were “coached” throughout a year by “university-based”, experienced literacy educators (Outlaw & Grifenhagen, 2021). The process of coaching consisted of literacy educators travelling to “novice” teachers’ “classrooms at least once per month to engage in active … modelling, co-teaching, and co-planning literacy instruction” (p. 249). The key feature of “coaching” highlighted in this, and other studies (Chambers Cantrell & Hughes, 2008; Ciampa & Gallagher, 2016; Loughland & Ryan, 2022; Tilton & Hartnett, 2016; Wang et al., 2017) appears to be the process of modelling. For the teachers in our study, being mentored by their colleagues and school leaders through modelling, and receiving helpful advice and resources, was a naturalistic source of growth in their self-efficacy.

Conversely, we found that a teacher’s self-efficacy in teaching reading can be undermined and *weakened* by a teacher being disrespected by a school leader, which may even lead to them experiencing psychological distress. Teachers’ self-efficacy can also be weakened by lack of explanation from school leaders about the rationale for implementing a new systematic strategy or programme for teaching reading.

**Fig. 1:**
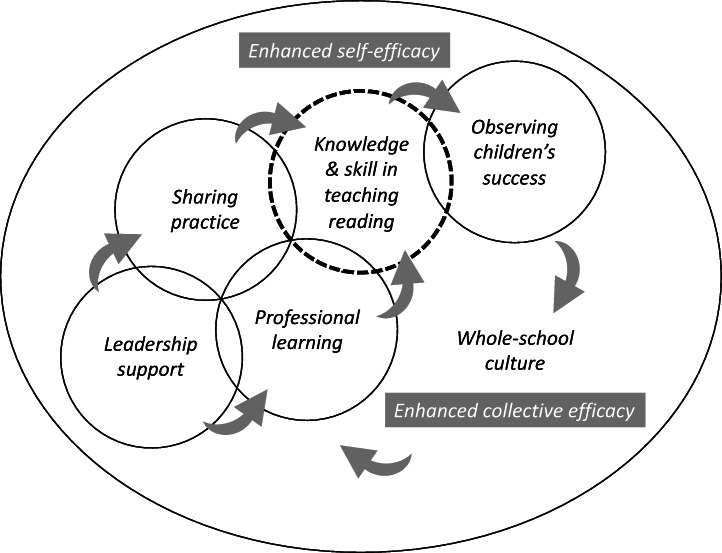
Model of interaction of sources of growth in teachers’ efficacy in teaching reading

Overall, our study shows that teachers’ collective efficacy in teaching reading is enhanced when all the sources of growth in self-efficacy referred to above come together in a whole-school, collaborative culture. This whole-school culture is characterised by leadership support for professional learning and implementation of new reading programmes, and colleagues sharing practice by modelling, leading to all teachers feeling safe to try new strategies and having mastery experiences in teaching reading, and seeing the children across the school being successful in reading as a result of their collaborative efforts. In such a school culture, teachers come to believe that the school as a whole is successful in teaching children to read. This combination and interaction of sources of growth in teachers’ efficacy in teaching reading is depicted in the model in Fig. 1.

The overlapping circles in the model are intended to illustrate the interaction between naturalistic sources of growth in teachers’ efficacy in teaching reading. Teachers must have some minimum level of knowledge and skill in literacy instruction for them to even begin trying to teach children to read. Teachers’ knowledge and skill is represented by the bold dashed circle, with the dashes indicating that their knowledge and skill continues to deepen and broaden throughout their careers as they engage with experienced colleagues and professional learning opportunities, e.g. about new research- and evidence-informed reading programmes.

Our results confirm predictions about sources of collective efficacy that include evidence of children’s success, and school leaders’ respect for and trust in teachers and support for their collaboration (Donohoo et al., 2018). However, whereas Donohoo et al. emphasise “learning” or test data as “evidence of impact”, we also found that teachers’ own, naturalistic and authentic experience in watching and hearing children read, and seeing them enjoy their reading etc., is an important source of evidence just as valid as test data.

#### Implications

We believe our model of teacher efficacy can inform decision-making in schools about ways to support and enhance teachers’ self- and collective efficacy in teaching reading. Specifically, school leaders could encourage and support their teachers to share—and could lead by example in modelling—successful practice. Leading by example is a key characteristic of principals’ practice in successful schools (Jacobson et al., 2005). School leaders could also support teachers’ professional learning, e.g. by enabling teachers to relate a new reading programme to their current practices and giving them time and trusting them to master new strategies. When a new reading programme includes ongoing support from external facilitators or “coaches”, we believe these coaches need to acknowledge and complement the collaborative culture of sharing practice that may already exist in a school.

The limitations of this study are that the small sample of teachers limits the extent to which our findings can be generalised. Teachers who volunteered to participate came from a select number of five relatively small schools in western NSW. The size of these schools and their western rural locations, where families and communities often face and overcome adversity, may mean that teachers in these schools are more resilient and prepared to work collaboratively in their teaching. As one teacher in our study commented, “generally, Bush people just get on with it. We just do what you have to do”. It may be that teachers from schools in other types of rural (e.g. coastal) areas, larger schools in regional and rural NSW, or schools within the Sydney metropolitan region, would have different views compared to our participants. However, our results are corroborated by findings from several areas of previous research on teacher efficacy, which suggests they do have validity.

Future research could focus on testing the validity of our themes and predictions of our model by undertaking a survey of primary teachers across all of regional and rural NSW, as well as the Sydney metropolitan area. Research could also be undertaken to evaluate strategies and initiatives that will support school leaders in taking a whole-school approach to supporting their teachers in helping children to be successful in reading.

#### Conclusion

Teachers’ self- and collective efficacy are important factors in determining the effort they make and how resilient they are in the face of challenges in helping children learn to read. Our study adds to research in this domain of teacher efficacy by exploring how teachers themselves perceive their efficacy is strengthened in natural ways through their professional practice. Teachers experience growth in their efficacy through observing children’s success and engagement in reading, receiving support from peers and school leaders through advice and modelling, and being respected, encouraged, and trusted in their capability to implement new practices by school leaders. Teachers also experience growth in their efficacy through professional learning in which they develop new knowledge and skill for teaching reading that enables them to be successful. Teachers’ collective efficacy or beliefs about their school to be successful is strengthened when all these sources combine in a collaborative, whole-school culture in which teachers feel safe to try new, effective practices. Well-planned and sustained support for developing supportive and collaborative whole-school cultures can potentially help to enhance all children’s achievement in reading.

## Data Availability

All audio recordings, interview transcripts, and digital/PDF files of scanned hard copy materials are stored in a project file repository in the University of Sydney’s Research Electronic Data Capture (REDCap) secure web application. Only the authors have password-protected access to the stored data in REDCap.
